# An indirect comparison of 144-week efficacy, safety, and tolerability of dolutegravir plus lamivudine and second-generation integrase inhibitor–based, 3-drug, single-tablet regimens in therapy-naive people with HIV-1

**DOI:** 10.1186/s12981-023-00507-1

**Published:** 2023-03-22

**Authors:** Lee A. Evitt, Sakina Nanji, Richard A. Grove, Chinyere Okoli, Jean van Wyk, Sonya J. Snedecor

**Affiliations:** 1 GSK, 980 Great West Road, Brentford, Middlesex, TW8 9GS UK; 2 OPEN Health, Bethesda, MD USA; 3 ViiV Healthcare, Brentford, UK

**Keywords:** Antiretroviral agents, HIV-1 infection, Dolutegravir, Lamivudine, Bictegravir, Emtricitabine, Tenofovir alafenamide

## Abstract

**Background:**

The long-term efficacy and safety of the 2-drug regimen dolutegravir (DTG) + lamivudine (3TC) and 3-drug single-tablet regimens recommended for antiretroviral therapy (ART)-naive people with HIV-1 (PWH) have yet to be compared directly in clinical trials. This indirect treatment comparison (ITC) was conducted to compare the durability of efficacy and long-term safety of DTG + 3TC vs second-generation, integrase strand transfer inhibitor (INSTI)-based, 3-drug, single-tablet regimens bictegravir/emtricitabine/tenofovir alafenamide (BIC/FTC/TAF) and DTG/abacavir/3TC (DTG/ABC/3TC) at Week 144 after treatment initiation.

**Methods:**

A systematic literature review identified 4 trials evaluating the treatment regimens of interest in ART-naive PWH (GEMINI-1, GEMINI-2, GS-US-380-1489, and GS-US-380-1490). Safety, efficacy, and tolerability results were compared using fixed-effects Bucher ITC methodology to calculate relative outcomes.

**Results:**

Rates of virologic suppression (HIV-1 RNA < 50 copies/mL, US Food and Drug Administration Snapshot analysis) and virologic failure (HIV-1 RNA ≥ 50 copies/mL) as well as mean change in CD4 + cell count were similar with DTG + 3TC, BIC/FTC/TAF, and DTG/ABC/3TC at Week 144. Serious adverse events occurred less frequently with DTG + 3TC compared with both BIC/FTC/TAF (odds ratio [OR], 0.51; 95% CI 0.29–0.87; *P* = 0.014) and DTG/ABC/3TC (OR, 0.38; 95% CI 0.19–0.75; *P* = 0.006). Discontinuations and overall adverse events were similar across all 3 regimens.

**Conclusions:**

These results suggest that the 2-drug regimen DTG + 3TC offers comparable and durable efficacy with fewer serious adverse events vs BIC/FTC/TAF and DTG/ABC/3TC through 144 weeks of treatment in ART-naive PWH. These long-term comparative data support the therapeutic value of DTG + 3TC for PWH.

**Supplementary Information:**

The online version contains supplementary material available at 10.1186/s12981-023-00507-1.

## Introduction

HIV treatment guidelines published by the US Department of Health and Human Services (DHHS) in 2021, the International Antiviral Society–USA (IAS) in 2020, and the European AIDS Clinical Society (EACS) in 2020 recommend integrase strand transfer inhibitor (INSTI)-based antiretroviral therapy (ART) with either 1 or 2 nucleoside reverse transcriptase inhibitors for most previously untreated adults and adolescents [1–3]. Considering the requirement for lifelong ART, the high prevalence of comorbidities among people with HIV (PWH), and the toxicities associated with ART, 2-drug regimens that maintain efficacy comparable to that of 3-drug regimens are of considerable interest and potential value, offering the possibility of reduced cumulative drug exposure, adverse effects, and potential drug interactions [1, 2, 4]. The 2-drug regimen dolutegravir (DTG) + lamivudine (3TC) demonstrated non-inferior efficacy, a high barrier to resistance, and a comparable safety profile relative to the 3-drug regimen DTG + tenofovir disoproxil fumarate/emtricitabine (TDF/FTC) at 144 weeks after treatment initiation in the GEMINI-1 and GEMINI-2 trials in ART-naive PWH [5]. Notably, there was a lower risk of drug-related adverse events (AEs) through Week 144 with DTG + 3TC than DTG + TDF/FTC in the pooled safety population from these 2 studies [5]. Efficacy and safety of the recommended 3-drug ART regimens have also been well established [1, 2]. Although DTG in combination with 3TC has been directly compared with DTG + TDF/FTC in ART-naive PWH, this 2-drug regimen has not been directly compared with other second-generation INSTI-based, 3-drug combinations in randomized clinical trials, ie, those containing bictegravir [5]. In the absence of randomized clinical trial data, indirect treatment comparisons can provide valuable supplementary information for clinicians, PWH, and other interested parties. Previous network meta-analyses have shown DTG + 3TC to have efficacy and safety comparable to those of guideline-recommended 3-drug regimens at Weeks 48 and 96 [6, 7].

This indirect treatment comparison was undertaken to further assess the durability of efficacy and long-term safety of DTG + 3TC vs recommended second-generation, INSTI-based regimens 144 weeks after treatment initiation.

## Methods

### Study identification

Systematic literature searches of PubMed, Embase, and Cochrane databases were conducted in 2013, 2018, and 2019, as previously described [6–9]. The aim of these searches was to identify phase 3 or 4 randomized controlled trials evaluating the efficacy and/or safety outcomes of regimens recommended by DHHS or EACS guidelines in treatment-naive adult or adolescent (aged ≥ 13 years) PWH [1, 2, 6, 9]. Identified trials evaluating second-generation guideline-recommended INSTI-based ART regimens were described in previously published meta-analyses based on Week 48 and Week 96 results [6, 8]. A manual search was subsequently conducted to identify any reported 144-week outcomes from the same clinical trials. Internal clinical study reports were used for unpublished data from ViiV studies.

### Outcomes

Efficacy outcomes of interest in the indirect treatment comparison were the proportion of trial participants achieving virologic suppression (HIV-1 RNA < 50 copies/mL) calculated according to the US Food and Drug Administration (FDA)-defined Snapshot algorithm [10] and the proportion with virologic failure (HIV-1 RNA ≥ 50 copies/mL; missing, switch, or discontinuation equals failure). CD4 + cell counts reported as mean change from baseline in the number of cells per microliter were also included for analysis.

All-cause discontinuations, discontinuations due to AEs, grade 3 or 4 AEs, serious AEs, and drug-related AEs through 144 weeks of treatment were included in the comparison of safety and tolerability.

Otherwise identical regimens with either TDF or TAF were assumed to have no clinical differences in efficacy, safety, and tolerability outcomes, which is supported by a meta-analysis showing no significant differences between TAF/FTC and TDF/FTC for these endpoints [11]. Therefore, DTG + TDF/FTC was assumed to be equivalent to DTG + TAF/FTC in this analysis.

### Statistical analysis

Analyses were performed using Microsoft Excel (Redmond, WA). Anchored Bucher’s frequentist-adjusted, fixed-effects indirect treatment comparison methodology [12] was used to evaluate relative outcomes between treatments, in accordance with the International Society for Pharmacoeconomics and Outcomes Research guidelines [13]. This method indirectly compares the effect of 2 therapies when the randomized controlled trials evaluating them both share a common control arm. The results were expressed as risk difference for virologic outcomes, mean difference for change in CD4 + cell count, and odds ratio (OR) with 95% CIs for safety outcomes. Indirect treatment comparison–generated risk differences and mean differences with 95% CIs that did not contain 0, and ORs with 95% CIs that did not contain 1, were considered statistically significant.

## Results

### Study characteristics

**Fig. 1:**
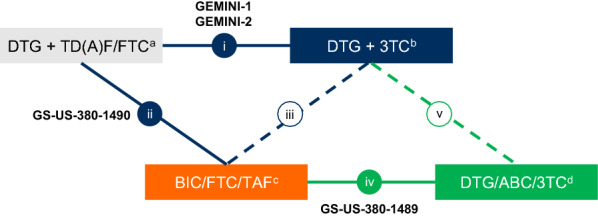
Schematic of studies and treatment regimens included in the indirect treatment comparison. Data from direct comparisons (solid lines and filled circles) between (i) DTG + 3TC and DTG + TD(A)F/FTC (GEMINI-1 and GEMINI-2) and (ii) BIC/FTC/TAF and DTG + TD(A)F/FTC (GS-US-380–1490) were used for indirect comparison (dashed lines and open circles) between (iii) DTG + 3TC and BIC/FTC/TAF. Data from indirect comparison (iii) and direct comparison between (iv) BIC/FTC/TAF and DTG/ABC/3TC (GS-US-380–1489) were used for indirect comparison between (v) DTG + 3TC and DTG/ABC/3TC. ABC, abacavir; BIC, bictegravir; DTG, dolutegravir; FTC, emtricitabine; QD, once daily; TAF, tenofovir alafenamide; 3TC, lamivudine; TDF, tenofovir disoproxil fumarate. ^a^DTG 50 mg + FTC 200 mg/TDF 300 mg QD or TAF 25 mg QD (DTG + TDF/FTC was assumed to be clinically equivalent to DTG + TAF/FTC). ^b^DTG 50 mg + 3TC 300 mg QD. ^c^BIC 50 mg/FTC 200 mg/TAF 25 mg QD. ^d^DTG 50 mg/ABC 600 mg/3TC 300 mg QD

Four studies were identified for inclusion (GEMINI-1, GEMINI-2, GS-US-380–1489, and GS-US-380-1490; Additional file 1: Table S1) [5, 14, 15], all of which have been previously included in published indirect treatment comparisons [6–8, 16–18]. The 4 studies formed a connected network via DTG + TD(A)F/FTC and bictegravir/FTC/tenofovir alafenamide (BIC/FTC/TAF; Fig. 1). Our analysis included a 2-step process: DTG + 3TC was initially indirectly compared with BIC/FTC/TAF based on DTG + TD(A)F/FTC being the common comparator to each regimen in the GEMINI trials and the GS-US-380-1490 trial, respectively. This result was subsequently used to indirectly compare DTG + 3TC with DTG/ABC/3TC using BIC/FTC/TAF (GS-US-380–1489 trial) as a common comparator (Fig. 1).

All 4 included studies were randomized, active-controlled, multinational trials that enrolled ART-naive adults. Eligible participants had HIV-1 RNA ≥ 500 copies/mL (GS-US-380-1489 and GS-US-380-1490) [14, 15] or 1000 to 500,000 copies/mL (GEMINI-1 and GEMINI-2) [5] and showed no evidence of viral resistance mutations to study drugs. Participants in GS-US-380-1489 and GS-US-380-1490 were required to have an estimated glomerular filtration rate ≥ 30 mL/min [14, 19]. In GS-US-380-1489, participants who were HLA-B*5701 positive or who had hepatitis B virus were excluded [14]. In GS-US-380-1490, participants with hepatitis B or C virus and previous antiretroviral use for pre-exposure or post-exposure HIV prophylaxis were permitted [19]. Women of reproductive potential were eligible for the GEMINI trials if they were not pregnant or lactating and using highly effective contraception, and exclusion criteria included active Centers for Disease Control and Prevention stage 3 HIV disease except for cutaneous Kaposi’s sarcoma and CD4 + cell count < 200 cells/μL [20].

Consistent with previous publications [6–8, 16–18], despite some small differences in inclusion and exclusion criteria, we found that the trial populations were similar with respect to key baseline characteristics, including age, sex at birth, race and ethnicity, mean viral load (4.39 to 4.45 HIV-1 RNA log_10_, copies/mL), and mean CD4 + cell count (453 to 476 cells/μL; Additional file 1: Table S2).

### Efficacy

**Fig. 2:**
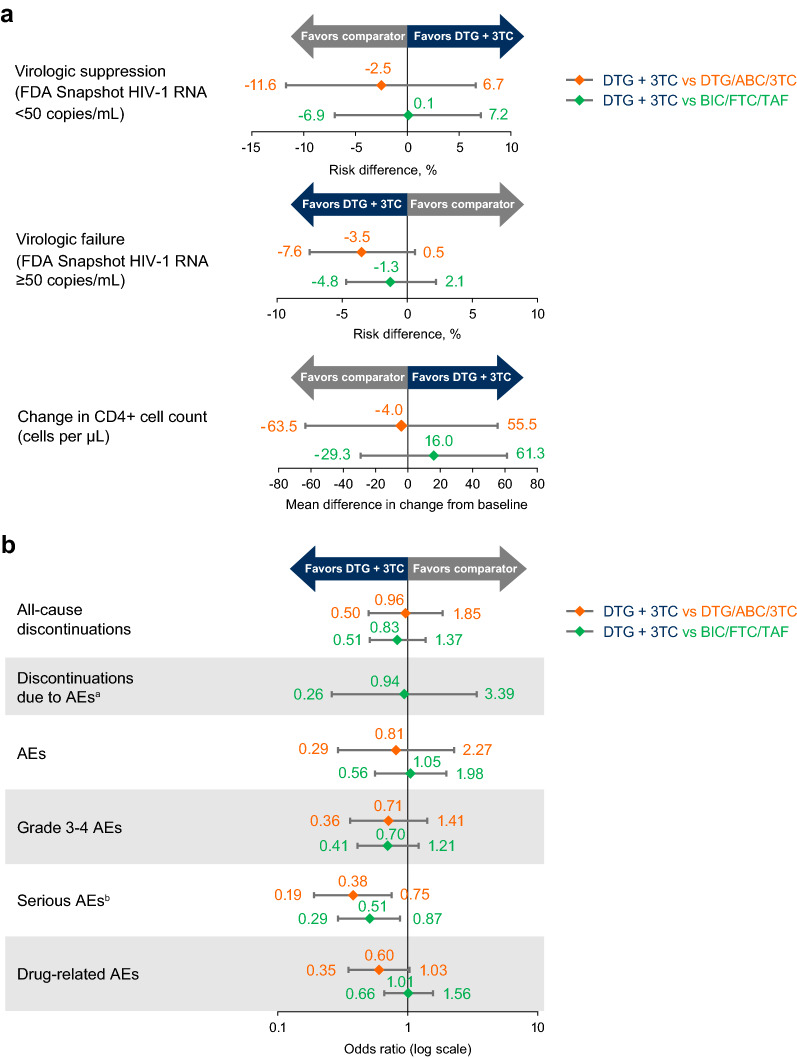
**a** Efficacy and **b** safety results of the indirect treatment comparisons at Week 144. Comparisons assumed TDF/FTC and TAF/FTC to be equivalent. Horizontal lines represent 95% CI. ABC, abacavir; BIC, bictegravir; DTG, dolutegravir; FDA, US Food and Drug Administration; FTC, emtricitabine; TAF, tenofovir alafenamide; 3TC, lamivudine. ^a^Comparison against DTG/ABC/3TC could not be estimated due to zero events in one treatment group. ^b^Fewer SAEs occurred with DTG + 3TC than with BIC/FTC/TAF (*P* = 0.014) and DTG/ABC/3TC (*P* = 0.006)

For the different treatment groups of the individual trials included in the analysis, the percentage of participants with virologic suppression ranged from 81.5% to 84.1% and the percentage with virologic failure ranged from 0.6% to 4.7% (Table 1). The results of the indirect treatment comparison showed no difference between DTG + 3TC and the 3-drug INSTI-based regimens BIC/FTC/TAF or DTG/ABC/3TC based on the risk difference (95% CI) for Week 144 virologic suppression (0.1% [− 6.9%, 7.2%] and − 2.5% [− 11.6%, 6.7%], respectively) and virologic failure (− 1.3% [− 4.8%, 2.1%] and − 3.5% [− 7.6%, 0.5%], respectively; Fig. 2 and Additional file 1: Table S3). Mean changes from baseline to Week 144 in CD4 + cell count were also similar for all 4 treatment regimens ranging from 278 to 317 cells/μL (Table 1) and in the indirect treatment comparison (Fig. 2 and Additional file 1: Table S3).

**Table 1:** Week 144 efficacy and safety data from the included trials

Outcome, n (%)^a^	GEMINI-1/-2 (pooled analysis)		GS-US-380-1489		GS-US-380-1490	
	DTG + 3TC (N = 716)	DTG + TDF/FTC (N = 717)	BIC/FTC/TAF (N = 314)	DTG/ABC/3TC (N = 315)	DTG + TAF/FTC (N = 325)	BIC/FTC/TAF (N = 320)
HIV-1 RNA < 50 copies/mL^b^	584 (81.6)	599 (83.5)	256 (81.5)	265 (84.1)	273 (84.0)	262 (81.9)
HIV-1 RNA ≥ 50 copies/mL^b^	23 (3.2)	21 (2.9)	2 (0.6)	9 (2.9)	10 (3.1)	15 (4.7)
Change in CD4 + cell count from baseline, mean (SD), cells/µL	300 (203.5)	298 (227.1)	299 (224.9)	317 (219.5)	289 (218.5)	278 (236.6)
Discontinuations	134 (18.7)	123 (17.2)	54 (17.2)	48 (15.2)	47 (14.5)	59 (18.4)
AEs	613 (85.6)	625 (87.2)	300 (95.5)	304 (96.5)	300 (92.3)	291 (90.9)
Grade 3–4 AEs	83 (11.6)	88 (12.3)	50 (15.9)	50 (15.9)	43 (13.2)	54 (16.9)
Serious AEs	76 (10.6)	85 (11.9)	41 (13.1)	53 (16.8)	40 (12.3)	63 (19.7)
Drug-related AEs	146 (20.4)	192 (26.8)	94 (29.9)	132 (41.9)	95 (29.2)	71 (22.2)
Discontinuations due to AEs	24 (3.4)	25 (3.5)	0 (0)	5 (1.6)	6 (1.8)	6 (1.9)

ABC, abacavir; AE, adverse event; BIC, bictegravir; DTG, dolutegravir; FDA, US Food and Drug Administration; FTC, emtricitabine; TAF, tenofovir alafenamide; 3TC, lamivudine; TDF, tenofovir disoproxil fumarate

^a^Unless otherwise specified. ^b^FDA Snapshot algorithm

### Safety

Serious AEs occurred less frequently in the DTG + 3TC treatment group than with the 3-drug regimens (Table 1). Among those treated with DTG + 3TC, the odds of experiencing a serious AE were lower than those treated with BIC/FTC/TAF (OR [95% CI], 0.51 [0.29–0.87]; *P* = 0.014) or DTG/ABC/3TC (OR [95% CI], 0.38 [0.19–0.75]; *P* = 0.006; Fig. 2).

The frequencies of discontinuations (all-cause and AE-related) and AEs (all-cause, drug-related, and grade 3 or 4) were similar between DTG + 3TC and the comparator regimens (Fig. 2 and Additional file 1: Table S3). A comparison of DTG + 3TC and DTG/ABC/3TC was not possible due to the lack of AE-related discontinuations in the BIC/FTC/TAF group of the GS-US-380-1489 trial.

## Discussion

Guideline-recommended initial ART regimens for most PWH include INSTI-based 3-drug and 2-drug regimens that have “demonstrated durable virologic efficacy, favorable tolerability and toxicity profiles, and ease of use” but have not been compared with each other in randomized clinical trials in ART-naive PWH [1, 2]. In the absence of randomized data, indirect treatment comparisons can provide useful information to help clinicians, PWH, and other interested parties, such as payers, make appropriate treatment choices. Long-term data are particularly relevant because PWH require lifelong ART to maintain virologic suppression. In addition, the high prevalence of comorbidities associated with HIV, especially in older adults, means that safety and tolerability are important to consider [1, 2].

The findings of our indirect treatment comparison suggest that DTG + 3TC offers similar efficacy (measured by virologic suppression and change from baseline in CD4 + cell count), with a comparable or better safety profile than BIC/FTC/TAF and DTG/ABC/3TC at Week 144 in previously untreated adults and adolescents. In particular, DTG + 3TC was estimated to result in fewer serious AEs than BIC/FTC/TAF and DTG/ABC/3TC. These results are broadly in line with results of analyses carried out at earlier time points (48 and 96 weeks) and add long-term data to the growing body of evidence supporting the non-inferiority of 2-drug regimens compared with 3-drug regimens [7, 9].

In GS-US-380-1489 and GS-US-380-1490, no participants had treatment-emergence resistance [14]. In GEMINI-1 and -2, no participants who met confirmed virologic withdrawal criteria had treatment-emergent resistance [5]. One participant with reported non-adherence in the GEMINI trials had treatment-emergent R263R/K at Week 144, which conferred a 1.8-fold reduction in susceptibility to DTG [5]. Altogether, these findings support the high barrier to resistance of standard-of-care INSTI-based regimens through 3 years.

Larger, more complex networks of randomized controlled trials are usually analyzed using Bayesian methodology [6–8, 16, 18], whereas the structure of our network allowed the use of the simpler Bucher analysis. This method has been widely used in various therapeutic areas and has not been shown to generate generally consistent results in similarly structured networks [21–25]. All the trials included in the analysis were designed to demonstrate anticipated equivalence between the treatment regimens.

Few long-term randomized clinical trial data exist for guideline-recommended ART regimens as many trials switch to open-label designs after 48 or 96 weeks. Albeit limited, the trials included in our analysis are landmark investigations of the respective treatment regimens and all remained blinded through Week 96 (GEMINI-1 and GEMINI-2) or 144 (GS-US-380-1489 and GS-US-380-1490) and included pre-specified secondary endpoints at Week 144 [5, 14, 15]. We are unaware of any controversy surrounding the validity of the findings from these trials, and we consider that the comparative estimates from this work are derived from the best evidence currently available.

In conclusion, the results of this indirect treatment comparison suggest that DTG + 3TC offers comparable and durable efficacy with fewer serious AEs vs BIC/FTC/TAF and DTG/ABC/3TC at Week 144 in ART-naive PWH. These long-term comparative data support the therapeutic value of the 2-drug regimen DTG + 3TC as first-line treatment for PWH.

## Supplementary Information

**Additional file 1: Table S1.** Summary of Study Designs of the Included Trials. Table detailing the study designs of the trials included in the analysis. **Table S2.** Summary of Baseline Characteristics of the Included Trials. Table showing baseline characteristics for the study populations included in the analysis. **Table S3.** Results of the Indirect Treatment Comparison for DTG + 3TC vs BIC/FTC/TAF and DTG/ABC/3TC at Week 144. Table showing odds ratios, risk differences, and mean differences from the indirect treatment comparison at Week 144.

## Data Availability

All data analyzed during this study are included in this published article and its supplementary information files.

## Abbreviations

ABC: Abacavir
AE: Adverse event
ART: Antiretroviral therapy
BIC: Bictegravir
DHHS: Department of Health and Human Services
DTG: Dolutegravir
EACS: European AIDS Clinical Society
FDA: US Food and Drug Administration
FTC: Emtricitabine
IAS: International AIDS Society–USA
INSTI: Integrase strand transfer inhibitor
ITC: Indirect treatment comparison
OR: Odds ratio
PWH: People with HIV-1
TAF: Tenofovir alafenamide
3TC: Lamivudine
TDF: Tenofovir disoproxil fumarate
