# Supplementary material for: An indirect comparison of 144-week efficacy, safety, and tolerability of dolutegravir plus lamivudine and second-generation integrase inhibitor–based, 3-drug, single-tablet regimens in therapy-naive people with HIV-1
Source: AIDS Res Ther. 2023 Mar 22;20:17. doi: 10.1186/s12981-023-00507-1 (PMC10031916; doi:10.1186/s12981-023-00507-1)
Supplement: Supplementary file 1 — Additional file 1: Table S1. Summary of Study Designs of the Included Trials. Table detailing the study designs of the trials included in the analysis. Table S2. Summary of Baseline Characteristics of the Included Trials. Table showing baseline characteristics for the study populations included in the analysis. Table S3. Results of the Indirect Treatment Comparison for DTG + 3TC vs BIC/FTC/TAF and DTG/ABC/3TC at Week 144. Table showing odds ratios, risk differences, and mean differences from the indirect treatment comparison at Week 144. [file 12981_2023_507_MOESM1_ESM.pdf]

ADDITIONAL FILE 1

Table S1. Summary of Study Designs of the Included Trials

**Trial name (ClinicalTrials.gov identifier)**	**Dosing regimen**	**Primary outcome**
GEMINI-1 and GEMINI-2 (NCT02831673 and NCT02831764)	DTG 50 mg + 3TC 300 mg QD<br>OR<br>DTG 50 mg + TDF 300 mg/FTC 200 mg QD	HIV-1 RNA <50 copies/mL (FDA Snapshot algorithm) at Week 48 in the ITT-E population
GS-US-380-1489 (NCT02607930)	BIC 50 mg/FTC 200 mg/TAF 25 mg QD<br>OR<br>DTG 50 mg/ABC 600 mg/3TC 300 mg QD	HIV-1 RNA <50 copies/mL (FDA Snapshot algorithm) at Week 48
GS-US-380-1490 (NCT02607956)	BIC 50 mg/FTC 200 mg/TAF 25 mg QD<br>OR<br>DTG 50 mg + TAF 25 mg/FTC 200 mg QD	HIV-1 RNA <50 copies/mL (FDA Snapshot algorithm) at Week 48

ABC, abacavir; BIC, bictegravir; DTG, dolutegravir; FDA, US Food and Drug Administration; FTC, emtricitabine; ITT-E, intention-to-treat–exposed; QD, once daily; TAF, tenofovir alafenamide; 3TC, lamivudine; TDF, tenofovir disoproxil fumarate.

Table S2. Summary of Baseline Characteristics of the Included Trials

	GEMINI-1/-2 (pooled analysis)		GS-US-380-1489		GS-US-380-1490	
**Baseline parameter, n (%)[a]**	**DTG + 3TC (N=716)**	**DTG + TDF/FTC (N=717)**	**BIC/FTC/TAF (N=314)**	**DTG/ABC/3TC (N=315)**	**DTG + TAF/FTC (N=325)**	**BIC/FTC/TAF (N=320)**
Median age, y	32	33	31	32	34	33
Male (sex at birth)	603 (84)	619 (86)	285 (91)	282 (90)	288 (89)	280 (88)
Race						
White	484 (68)	499 (70)	180 (57)	179 (57)	195 (60)	183 (57)
Black or African American	90 (13)	71 (10)	114 (36)	112 (36)	100 (31)	97 (30)
Asian	71 (10)	72 (10)	6 (2)	10 (3)	10 (3)	7 (2)
Other	71 (10)	75 (10)	14 (4)	14 (4)	20 (6)	33 (10)
Ethnicity, Hispanic/Latinx	215 (30)	232 (32)	72 (23)	65 (21)	81 (25)	83 (26)
HIV-1 RNA $\log_{10}$, copies/mL, mean (SD)	4.42 (0.66)	4.45 (0.65)	4.41 (0.65)	4.42 (0.69)	4.42 (0.67)	4.39 (0.73)
Baseline CD4+ cell count, cells/µL, mean (SD)	462 (219.2)	461 (213.1)	453 (220.8)	476 (231.4)	454 (231.5)	457 (255.3)

ABC, abacavir; BIC, bictegravir; DTG, dolutegravir; FTC, emtricitabine; TAF, tenofovir alafenamide; 3TC, lamivudine; TDF, tenofovir disoproxil fumarate.

[a]Unless otherwise specified.

Table S3. Results of the Indirect Treatment Comparison for DTG + 3TC vs BIC/FTC/TAF and DTG/ABC/3TC at Week 144

**Comparison**	**DTG + 3TC vs BIC/FTC/TAF[a]**			**DTG + 3TC vs DTG/ABC/3TC[b]**		
**Comparative effect measure (95% CI)**	**Odds ratio**	**Risk difference**	**Mean change difference**	**Odds ratio**	**Risk difference**	**Mean change difference**
Virologic suppression (HIV-1 RNA <50 copies/mL)		0.1% (−6.9%, 7.2%)			−2.5% (−11.6%, 6.7%)	
Virologic failure (HIV-1 RNA ≥50 copies/mL)		−1.3% (−4.8%, 2.1%)			−3.5% (−7.6%, 0.5%)	
CD4+ cell count change from baseline, cells/µL			16.0 (−29.3, 61.3)			−4.0 (−63.5, 55.5)
All-cause discontinuations	0.83 (0.51, 1.37)			0.96 (0.50, 1.85)		
Discontinuations due to AEs	0.94 (0.26, 3.39)			Calculation not possible[c]		
All AEs	1.05 (0.56, 1.98)			0.81 (0.29, 2.27)		
Grade 3-4 AEs	0.70 (0.41, 1.21)			0.71 (0.36, 1.41)		
Serious AEs	**0.51 (0.29, 0.87)**			**0.38 (0.19, 0.75)**		
Drug-related AEs	1.01 (0.66, 1.56)			0.60 (0.35, 1.03)		

ABC, abacavir; AE, adverse event; BIC, bictegravir; DTG, dolutegravir; FTC, emtricitabine; TAF, tenofovir alafenamide; 3TC, lamivudine; TDF, tenofovir disoproxil fumarate.

[a]Indirect comparison based on data from direct comparisons between DTG + 3TC and DTG + TD(A)F/FTC (GEMINI-1 and GEMINI-2) and between BIC/FTC/TAF and DTG + TD(A)F/FTC (GS-US-380-1490). [b]Indirect comparison based on data from direct comparison between BIC/FTC/TAF and DTG/ABC/3TC (GS-US-380-1489) and indirect comparison between DTG + 3TC and BIC/FTC/TAF. [c]Comparison against DTG/ABC/3TC could not be estimated due to zero events in one treatment group.
